# *In vitro* parameter optimization for spatial control of focused ultrasound ablation when using low boiling point phase-change nanoemulsions

**DOI:** 10.1186/2050-5736-1-16

**Published:** 2013-09-13

**Authors:** Connor Puett, Linsey C Phillips, Paul S Sheeran, Paul A Dayton

**Affiliations:** 1 Joint Department of Biomedical Engineering, University of North Carolina at Chapel Hill and North Carolina State University, 109 Mason Farm Road, 304 Taylor Hall, CB 7575, Chapel Hill NC 27599, USA

**Keywords:** Phase-Shift, Perfluorocarbon, Vaporization, High-Intensity Focused Ultrasound (HIFU), Tissue ablation, Nanodroplet, Decafluorobutane

## Abstract

**Background:**

Phase-shift nanoemulsions (PSNEs) provide cavitation sites when the perfluorocarbon (PFC) nanodroplets (ND) are vaporized to microbubbles by acoustic energy. Their presence lowers the power required to ablate tissue by high-intensity focused ultrasound (HIFU), potentially making it a safer option for a broader range of treatment sites. However, spatial control over the ablation region can be problematic when cavitation is used to enhance heating. This study explored relationships between vaporization, ablation, and the PSNE concentration *in vitro* to optimize the acoustic intensity and insonation time required for spatially controlled ablation enhancement using a PSNE that included a volatile PFC component.

**Methods:**

HIFU (continuous wave at 1 MHz; insonation times of 5, 10, 15, and 20 s; cool-down times of 2, 4, and 6 s; peak negative pressures of 2, 3, and 4 MPa) was applied to albumin-acrylamide gels containing PFC agents (1:1 mix of volatile decafluorobutane and more stable dodecafluoropentane at 10^5^ to 10^8^ PFC ND per milliliter) or agent-free controls. Vaporization fields (microbubble clouds) were imaged by conventional ultrasound, and ablation lesions were measured directly by calipers. Controlled ablation was defined as the production of ‘cigar’-shaped lesions corresponding with the acoustic focal zone. This control was considered to be lost when ablation occurred in prefocal vaporization fields having a predominantly ‘tadpole’ or oblong shape.

**Results:**

Changes in the vaporization field shape and location occurred on a continuum with increasing PSNE concentration and acoustic intensity. Working with the maximum concentration-intensity combinations resulting in controlled ablation demonstrated a dose-responsive relationship between insonation time and volumes of both the vaporization fields (approximately 20 to 240 mm^3^) and the ablation lesions (1 to 135 mm^3^) within them.

**Conclusions:**

HIFU ablation was enhanced by this PSNE and could be achieved using intensities ≤650 W/cm^2^. Although the ablation lesions were located within much larger microbubble clouds, optimum insonation times and intensities could be selected to achieve an ablation lesion of desired size and location for a given PSNE concentration. This demonstration of controllable enhancement using a PSNE that contained a volatile PFC component is another step toward developing phase-shift nanotechnology as a potential clinical tool to improve HIFU.

## Background

High-intensity focused ultrasound (HIFU) is approved by the FDA in the USA for the treatment of uterine fibroids and is being evaluated for treating other kinds of tumors [1]. Ultrasound can kill cells by thermal effects (heating and denaturing proteins) and mechanical effects (disrupting cellular membranes). Both effects are enhanced if appropriately sized bubbles are present in the acoustic field [2,3]. Bubbles in an acoustic field oscillate (stable cavitation), absorbing ultrasound energy and releasing it as heat, and collapse (inertial cavitation), producing local shock waves [2]. Ultrasound can draw bubbles from the dissolved gases in biologic tissue. However, the required pressures [4] can result in the formation of bubble clusters or clouds with an unpredictable density and therefore an unpredictable enhancement of lesion formation. Hence, currently available HIFU systems employ acoustic parameters chosen to limit bubble formation. Without the assistance of cavitation, high intensities in the range of 2,000 W/cm^2^ are needed to reach ablative temperatures [5,6]. These intensities place healthy tissue in the ultrasound path at risk [7]. In order to avoid unwanted thermal damage, HIFU is delivered in short bursts separated by cool-down periods. The transrectal application of HIFU for prostate cancer employs 3 to 5 s of therapeutic insonation (‘on’ time), during which ablation occurs, followed by 5 to 6 s of cool-down (‘off’ time) [5,6]. Since each therapeutic burst results in an ablation lesion measuring 30 to 40 mm^3^, 2 to 5 h is typically required to ablate the total volume of prostate cancer. During this time, local or generalized anesthesia and tissue cooling are required. In response to these limitations, research is underway to minimize the energy required by HIFU to achieve tumor ablation. Enhanced focused ultrasound seeks to take advantage of cavitation, rather than avoid it, by seeding the acoustic field with bubbles.

Many studies to date have confirmed the ability of micron-sized bubbles or ‘microbubbles’ , including commercially available ultrasound contrast agents, to enhance HIFU in experimental models [3,8-12]. Enhancement refers to the faster production of larger ablation lesions using lower delivered energy. Ultrasound contrast agents are typically composed of high molecular weight gases, including volatile perfluorocarbons (PFC) such as octafluoropropane (C_3_F_8_, b.p. = -37°C), encapsulated in protein, polymer, or lipid shells [13]. These agents are designed to improve diagnostic imaging, and as such, they have short half-lives *in vivo* and are confined to the vasculature by their size [14]. Since they cannot penetrate into diseased tissue or remain in circulation for the time required to deliver therapeutic ultrasound, microbubbles have limited direct clinical utility in the setting of HIFU.

As a potential solution, PFC microbubbles can be generated in tissues by vaporizing liquid PFC nanodroplets (ND) using energy delivered by ultrasound [15]. Since the PFC core is encapsulated by surfactant, these particles are often referred to collectively as phase-shift nanoemulsions (PSNEs). PSNEs remain stable for hours at body temperature in solution and *in vivo*[16,17]. Additionally, droplets can be small enough (<300 nm) to extravasate through the leaky vasculature in malignant and inflamed tissues and have been shown to accumulate in tumors following their intravenous administration [17]. Therefore, PSNEs hold promise as potentially useful agents to enhance HIFU [15,18], and their behavior in response to HIFU has been studied in solution [16], in tissue-mimicking gels [9,19,20], and in animals [17].

However, control over ablation lesion geometry and placement can be problematic when the acoustic field contains PFC microbubbles or PSNEs. In an early trial of HIFU enhancement using octafluoropropane microbubbles in a tissue-mimicking gel, undesirable distortion in ablation lesions was noted with increasing bubble concentrations [11]. At lower bubble concentrations, the ablation lesions were ‘cigars’ (prolate ellipsoids, vertical axis > lateral axis) corresponding with the HIFU focal zone. As bubble concentrations increased, larger ablation lesions became ‘tadpoles’ , describing both their geometric appearance (asymmetric ellipsoids) and their relocation or migration away from the focal point and closer to the ultrasound source. Eventually, the ablation lesions flattened (oblate ellipsoids, lateral axis > vertical axis) along the frontal gel surface, completely ‘shadowing’ the desired focal point. The largest ablation lesions were therefore achieved at a price of lost control over lesion geometry and location. Similar control problems have also been encountered during HIFU enhancement with the use of liquid PFC droplets during phase-shift studies [9,20,21]. Using dodecafluoropentane (DDFP) droplets in an albumin-acrylamide gel model, Zhang P. et al. described ‘teardrop’ ablation lesions at higher intensities (>830 W/cm^2^) [20], while Zhang M. et al. reported complete loss of an ablation response at the highest microdroplet concentration [21]. The authors hypothesized that this loss resulted from ‘backscatter from the high-density acoustic droplet vaporization bubbles drawn proximally to shadow the focus’ [21].

Research is therefore being done to manipulate the HIFU acoustic parameters and PSNE concentrations to achieve controlled enhancement. The acoustic energy required to trigger the critical phase-shift from liquid droplet to microbubble is dependent on temperature, the frequency of insonation, the size of the droplet, and the type of PFC compound [15,22]. Smaller droplets are more difficult to vaporize than larger droplets, and volatile PFCs are easier to vaporize than less-volatile compounds. Most studies using phase-shift emulsions to enhance HIFU have employed PFC droplets containing dodecafluoropentane (C_5_F_12_, b.p. = 29°C), which has a boiling point close to body temperature [17,19-21]. As shown in a tissue-mimicking gel model, there is a large difference between the relatively high (approximately 8 MPa at a frequency of 2 MHz) peak negative pressure (PNP) required to vaporize DDFP droplets <500 nm in diameter and the much lower acoustic pressure needed to drive inertial cavitation once bubbles have been generated [19]. This difference provides an opportunity to control the otherwise unpredictable ablation response in the setting of cavitation. A brief (<1 ms) high-pressure pulse is used to localize vaporization, followed by continuous wave insonation at lower pressure to drive cavitation and achieve ablation.

Our laboratory has been exploring the possible diagnostic and therapeutic applications of PSNEs containing highly volatile PFC compounds, including decafluorobutane (DFB, C_4_F_10_, b.p. = -2°C) [23]. Blending PFC compounds to tune the vaporization threshold of PSNEs has been explored since its introduction in 2005 [24]. Previous experiments looking at HIFU enhancement using a PSNE containing a 1:1 mix of DFB and DDFP demonstrated vaporization within 10 to 20 ms of continuous wave HIFU (1 MHz) delivered at a pressure as low as 2 MPa. This low pressure was also adequate to drive cavitation and achieve ablation. Given this absence of a large difference between the pressures required for vaporization and cavitation, continuous wave HIFU was applied without a vaporization pulse [9]. The results defined the upper and lower nanodroplet concentration and acoustic pressure limits required to achieve enhanced ablation. Additionally, comparing the behavior of liquid nanodroplets and microbubbles directly demonstrated that droplet vaporization could be achieved focally, allowing for control over the location of ablation, although the ablation lesions were contained within larger vaporization fields of microbubbles.

The current study examined the effect of varying the insonation (‘on’) and cool-down (‘off’) times of the HIFU treatment cycle on the resulting microbubble clouds and ablation lesions within them over a range of PSNE concentrations and acoustic pressures. It is now clear that control over ablation during enhanced HIFU requires selecting the appropriate acoustic parameters for the available PFC concentration, a fact that is of particular importance when working with more volatile PSNEs. Zhang P. et al. have suggested that ‘there may potentially be an optimal range of acoustic intensities that allow for taking advantage of bubble-enhanced heating while avoiding distortion in lesion shape’ [20]. The goal of this study was therefore to optimize the acoustic intensity and insonation time for a given concentration of this unique PFC agent during continuous wave HIFU. The demonstration of controllable ablation in the presence of a volatile PFC component is another step toward developing PSNEs as useful clinical tools to enhance HIFU.

## Methods

### Preparation of the phase-shift perfluorocarbon nanoemulsion

The PSNE was prepared by the condensation procedure described previously [23]. Lipids (1,2-distearoyl-sn-glycero-3-phosphocholine and 1,2-distearoyl-sn-glycero-3-phosphoethanolamine-N-methoxy(polyethylene-glycol)-2000, purchased from Avanti Polar Lipids (Alabaster, AL, USA) in a 9:1 M ratio and a total lipid concentration of 1.0 mg/mL) were emulsified in a solution of propylene glycol 15% (*v*/*v*), glycerol 5% (*v*/*v*), and phosphate-buffered saline 80% (*v*/*v*). Then, 1.5 mL of the lipid mixture was placed in a sealed 3-mL glass vial and the air headspace exchanged with PFC gas (equal parts decafluorobutane (C_4_F_10_) and dodecafluoropentane (C_5_F_12_), purchased from Fluoromed (Round Rock, TX, USA)). Microbubbles with PFC cores (1:1 ratio of DFB and DDFP) and phospholipid shells formed spontaneously during agitation using a Vialmix Shaker (Bristol-Myers-Squibb, New York, NY, USA). The concentration of microbubbles in this emulsion was measured in triplicate using an Accusizer 780 (Particle Sizing Systems, Santa Barbara, CA, USA) and was found to be 10^10^ microbubbles per milliliter. Condensation of the microbubbles to nanodroplets was achieved by cooling the emulsion under an adjustable high-pressure air source in a CO_2_/isopropanol bath maintained at a temperature between -5°C and -10°C. The mixture was swirled gently while the headspace pressure in the vial was steadily increased until a change in the consistency of the solution indicated the onset of condensation. The droplets measured 240 ± 65 nm in diameter as determined by dynamic light scattering (Malvern Nano ZS, Malvern Instruments, Ltd., Westborough, MA, USA). Assuming a 1:1 conversion from microbubble to nanodroplet, a final PSNE stock solution containing 10^10^ PFC nanodroplets per milliliter was stored and used within 24 h.

### Tissue-mimicking phantom

**Figure 1:**
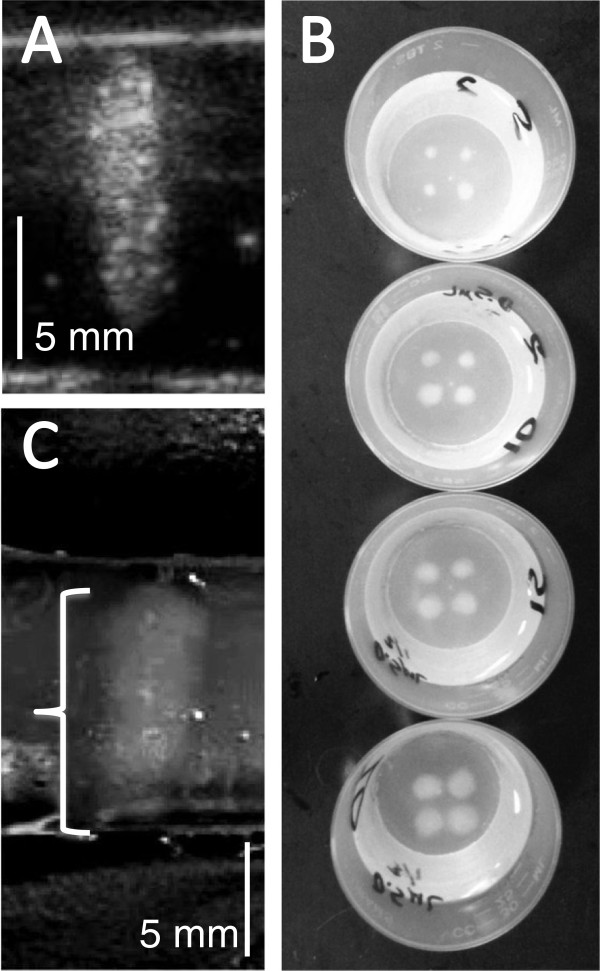
**Vaporization field (microbubble cloud) and ablation lesion images obtained after applying continuous wave HIFU.** HIFU was applied at 1 MHz to albumin-acrylamide gel phantoms containing 5 × 10^5^ PFC ND per milliliter. **(A)** This ultrasound image shows a cigar-shaped vaporization field generated after 20 s of HIFU at 650 W/cm^2^ (4 MPa). **(B)** These optical images show four opaque white ablation lesions located in each transparent gel phantom following the application of HIFU at 650 W/cm^2^ for 5, 10, 15 and 20 s. Larger lesions can be observed in phantoms treated with longer insonation times. **(C)** A single ablation lesion cut along its vertical axis, demonstrating the desired cigar shape.

Albumin-acrylamide tissue-mimicking phantoms were prepared and formed into 10-mL frustums (top diameter, 3 cm; bottom diameter, 4.1 cm; height, 1.2 cm) containing various concentrations of the stock PSNE [9,25]. The PSNE was gently stirred into the degassed acrylamide solution prior to polymerization in the frustum mold by the addition of 1% *v*/*v* of 10% ammonium persulfate and 0.4% *v*/*v* tetramethylethylenediamine. These albumin-acrylamide gels have acoustic properties similar to biologic tissues. Based on the final albumin concentration in these gels (35%), its density was 995 kg/m^3^, and sound traveled within the gel at a speed of 1,543 m/s [25]. In these studies, HIFU was delivered at 1 MHz, and at this frequency, attenuation by the gel was 0.28 dB/cm [25]. Vaporization fields (microbubble clouds) within the phantoms can be imaged by ultrasound (Figure 1A). Furthermore, the initially transparent gel turns opaque white in regions where the albumin is denatured at temperatures above 60°C, allowing for direct visual measurement of the ablation lesions (Figure 1B,C).

### Gel model of diseased tissue

A model for HIFU delivery was constructed as described previously in order to simulate a region of diseased tissue surrounded by healthy tissue [9]. The phantom containing the PFC nanoemulsion sat in a gellan gum mold [26] and was covered by a 10-mm-thick degassed, agent-free albumin-acrylamide gel cap. The mold and cap mimicked the healthy tissue surrounding the diseased phantom. Since they have similar acoustic impedances (albumin-acrylamide gel: 1.54 × 10^6^, gellan gum mold: 1.62 × 10^6^ kg/m^2^s) [25,26], significant reflection should not occur at the gel and mold interfaces. This *in vitro* model was designed to simulate PFC agent accumulation in a tumor. PSNE accumulation has been demonstrated *in vivo*[17] and is believed to result from the enhanced permeability of tumor vasculature [27]. The layered arrangement of gels allowed for assessment of ablation along the HIFU beam path and ensured that acoustic energies were low enough to avoid ablation in prefocal tissue regions containing no PFC agent. The tissue-mimicking gels and mold were maintained at a constant body temperature of 37°C by a bath circulating degassed water.

### Focused ultrasound

A spherically focused, eight-element, annular array transducer with active outer and inner diameters of 80 and 33.5 mm and a natural focal length of 80 mm (F# = 1) (Imasonic, Voray-sur-l’Ognon, France) was driven using a Therapy Imaging Probe System (TIPS) (Philips Research North America, Briarcliff Manor, NY, USA) to provide continuous wave HIFU at 1 MHz. The transducer was calibrated at 2, 3, and 4 MPa using a needle hydrophone (Onda HNA-0400, Sunnyvale, CA, USA) positioned at the focal point (TIPS focal zone: 1 × 1 × 6 mm) in a degassed water bath (free-field). Since measuring the acoustic intensity at the focal point directly in gels during HIFU is challenging, the intensities are estimates based on calculation using the free-field focal pressure, the acoustic impedance of the albumin-acrylamide gel, and the frequency-dependent attenuation by the gel. The nonlinear pressure waveform amplitudes recorded during calibration were derated for gel attenuation and averaged to obtain the focal pressure in the gel, which was squared and divided by the gel acoustic impedance (density × sound speed) to estimate the spatial peak intensity (*I*_SP_) in the focal zone during HIFU [28]. Intensities corresponding to PNPs of 2, 3, and 4 MPa were 140, 390, and 650 W/cm^2^, respectively. Previous experimentation has shown that (1) a PNP of 1 MPa (intensity = 30 W/cm^2^) does not generate a vaporization field or an ablation lesion across the nanodroplet concentration range used in this study, (2) both vaporization and ablation occur at a pressure of 2 MPa, demonstrating no large difference between the vaporization and cavitation pressure thresholds, and (3) agent-free (non-enhanced) ablation is just beginning to occur at a PNP of 4 MPa [9]. Acoustic intensities below 140 and above 650 W/cm^2^ were therefore not explored further. The HIFU treatment cycle was varied with insonation times of 5, 10, 15, and 20 s followed by cool-down times of 2, 4, and 6 s for each intensity. Four separate ablation lesions were generated in each phantom (Figure 1B). The same insonation time (5, 10, 15, or 20 s) was used to create each lesion in a single phantom. The cool-down time (2, 4, or 6 s) referred to the time separating each of the four insonations, during which time the HIFU focus was moved to a new position in the gel. The four focal zones in each phantom were separated by 8 mm. This separation distance was selected based on the results of previous testing [9] demonstrating that the diameters of the vaporization fields in the presence of this specific PSNE approach 8 mm at higher acoustic intensities and ND concentrations. Testing at each acoustic parameter was carried out at least eight times. The ultrasound transducer was positioned over the phantom at the focal length of 80 mm (64 mm through degassed water, 16 mm through albumin-acrylamide gel) and moved automatically by a programmable 3-D motion stage.

### Vaporization field shape and location

Liquid nanodroplets are only very weakly echogenic, since their density and compressibility are similar to surrounding tissue. However, the vaporization field (microbubble cloud) resulting from their phase-shift can be imaged by conventional ultrasound with high sensitivity [9]. In order to determine the maximum PSNE concentration allowable to produce an ablation lesion with the desired cigar shape centered at the focal point for a given acoustic intensity, continuous wave HIFU was applied for 20 s using PNPs of 2, 3, and 4 MPa to phantoms containing 0.1, 0.5, 1.0, 2.5, 10, 50, and 100 μL of the PSNE stock solution. The resulting nanodroplet concentrations in the phantoms were not measured directly, but assuming minimal PFC agent loss during preparation, they were calculated to be 1 × 10^5^, 5 × 10^5^, 1 × 10^6^, 2.5 ×10^6^, 1 × 10^7^, 5 × 10^7^, and 1 × 10^8^ ND per milliliter, respectively. Reported as a volume fraction (*v*/*v*) of the PSNE stock solution added to the 10 mL gel phantom, these concentrations were equivalent to a range from 0.001% to 1%. This broad range (10^5^ to 10^8^ ND per milliliter) was studied to document the behavior of this novel PSNE and to define the upper and lower PSNE concentration limits for effective HIFU enhancement. The lower concentrations are comparable to those used in other PFC microdroplet and nanodroplet phase-shift enhancement studies [20,21] and are more likely to be achievable *in vivo*.

**Figure 2:**
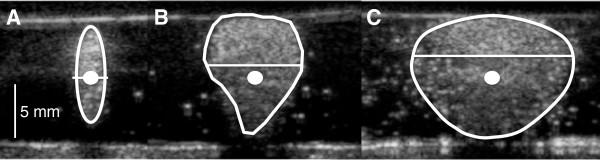
**Schematic representation of the changing shape of vaporization fields (microbubble clouds) with increasing PFC concentrations and acoustic energies. (A)** The desired shape is a cigar (prolate ellipsoid) centered at the acoustic focal point (white dot). **(B)** Tadpole lesions (asymmetric ellipsoids) occur as vaporization begins to concentrate in prefocal regions. **(C)** Eventually, the microbubble clouds flatten (oblate ellipsoids) and mass along the frontal phantom surface. These shape changes (distortion) can be appreciated qualitatively and can also be assessed quantitatively by measuring the length of the lateral axis (horizontal white line), the area-to-perimeter ratio, and the displacement of the centroid off of the focal plane (center shift).

The resulting vaporization fields were imaged using a 15 L8 transducer probe with a Siemens Sequoia scanner (Siemens, Mountain View, CA, USA) operating in B-mode (frequency = 14 MHz, mechanical index = 0.7). This imaging did not trigger PFC nanodroplet vaporization as confirmed by imaging phantoms prior to HIFU treatment. The microbubble cloud shapes and locations in the gel phantoms were analyzed using ImageJ software. Their lateral axis lengths, perimeters, and areas were measured and used to quantify the changing cloud shape (distortion). The progression from cigar to tadpole to oblate ellipsoid (Figure 2) can be demonstrated mathematically by a lengthening of the lateral axis and an increasing area-to-perimeter ratio. In order to quantify displacement of the microbubble cloud off the acoustic focus (center shift), the centroid of the cloud was calculated in ImageJ and its distance from the acoustic focus (assumed to be on the central plane of the phantom) was measured. Further testing of microbubble cloud and ablation lesion volumes in response to varied insonation and cool-down times was carried out using the maximum PSNE concentration for a given acoustic intensity that contained predominantly cigar-shaped ablation lesions centered at the acoustic focal point.

### Vaporization field and ablation lesion volumes

Previous testing has demonstrated that PFC-enhanced HIFU ablation lesions in the presence of a DFB-DDFP PSNE are located in larger vaporization fields (microbubble clouds) [9]. Knowing the extent of the vaporization field is useful when planning a strategy to position adjacent ablation lesions. A 15L8 transducer probe with a Siemens Sequoia scanner operating in B-mode at 14 MHz was used to image the vaporization field immediately after each HIFU treatment (Figure 1A). The PSNE concentration-intensity combinations identified for further study yielded vaporization fields characterized predominately as cigars when insonation times were less than 20 s. However, early tadpole transformation was present for some microbubble clouds after 20 s of insonation. Given this asymmetry, the volumes of all microbubble clouds were calculated using code written in Matlab. The code allowed a region of interest to be outlined (microbubble cloud perimeter) and sectioned horizontally. Three-dimensional disc volumes were calculated from these two-dimensional sections and the disc volumes summed for a total volume approximation. Ablation lesions were apparent as opaque white regions in the otherwise transparent phantom (Figure 1B,C). To determine the volume of these predominantly cigar-shaped lesions, the phantoms were cut by scalpel through the central vertical plane of the lesion (Figure 1C). The maximum height (*h*) and width (*w*) of these ablation lesions were measured by calipers, and the corresponding prolate ellipsoid volume (*V*) was calculated: *V* = (4/3) × π × (*h*/2) × (*w*/2)^2^.

### Statistical analysis

Means and corresponding standard deviations (S.D.) were reported and represented on the graphs, and *p* values of <0.05 (*α*) were considered to be statistically significant. One-way analysis of variance (ANOVA) was used to compare the means when more than two sample groups were present in the data set. A Bonferroni adjustment was made to the accepted level for statistical significance (*p* = *α*/*N*) in order to compare combinations of sample pairs (*N*) by Student's *t* test within the larger group.

## Results

### Vaporization field shape and location as a function of nanodroplet concentration and acoustic intensity

**Figure 3:**
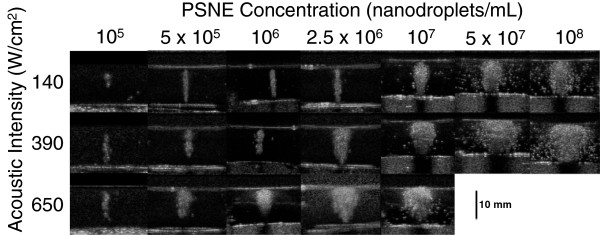
**Representative acoustic images of vaporization fields (microbubble clouds) generated by the application of continuous wave HIFU.** HIFU was applied at 1 MHz for 20 s at intensities of 140, 390, and 650 W/cm^2^ (PNP = 2, 3, and 4 MPa) to albumin-acrylamide phantoms containing PFC nanodroplets over a concentration range from 10^5^ to 10^8^ ND per milliliter. The progressive changes in the shape and location of the microbubble clouds with increasing PSNE concentrations can be seen. The shape change follows a predictable pattern from cigar (prolate ellipsoid) to tadpole (asymmetric ellipsoid) to oblate ellipsoid, as vaporization concentrates in prefocal regions.

**Figure 4:**
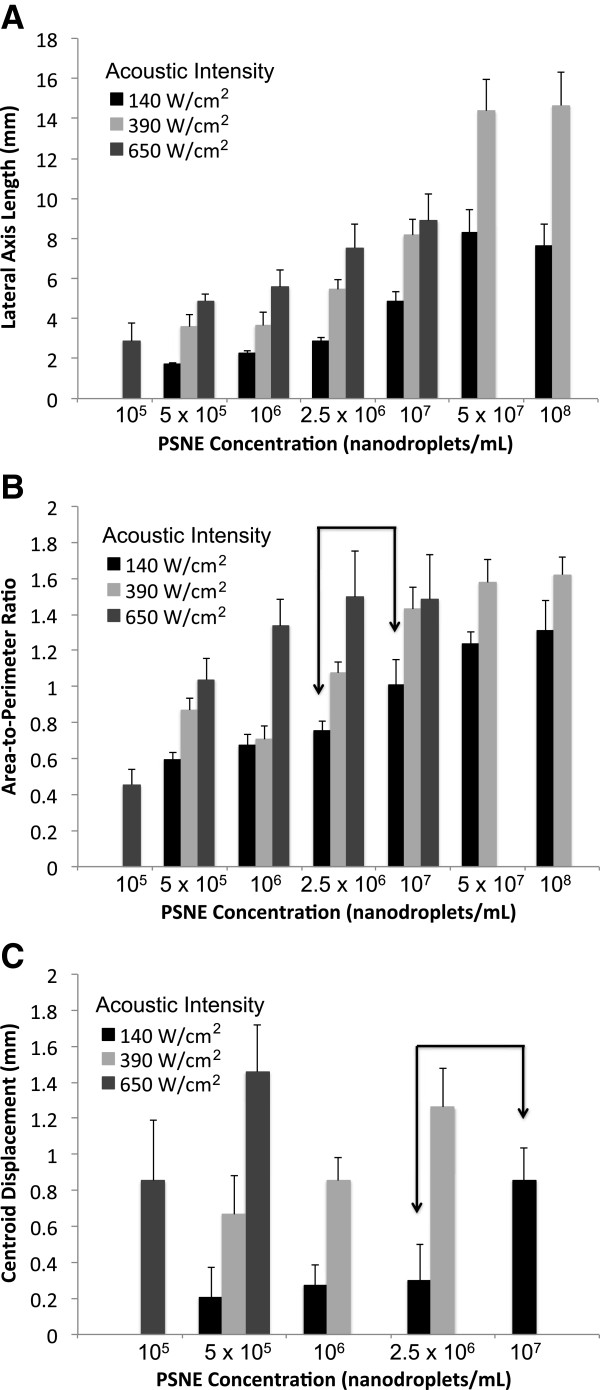
**Quantitative assessment of the progressive changes in the shape and location of the vaporization fields. (A)** There is a logarithmic relationship between the length of the lateral axis and the PSNE concentration for a given acoustic intensity (*R*^2^ ≥ 0.94, *p* < 0.001, linear regression on log scale), demonstrating that most of the change occurs in the lower PSNE concentration range. **(B)** Change in the area-to-perimeter ratio reflects changes in the microbubble cloud shape (distortion). **(C)** Prefocal displacement of the centroid (center shift) as a function of PSNE concentration. The first PSNE concentration for a given intensity to demonstrate a statistically significant increase in distortion compared to the previous concentration (*p* ≤ 0.01 by Student's *t* test) corresponded with the first concentration to demonstrate a statistically significant center shift (*p* ≤ 0.03 by Student's *t* test). These are the points of early tadpole transformation and occurred at PSNE concentrations of 5 × 10^5^, 2.5 × 10^6^, and 1 × 10^7^ ND per milliliter using intensities of 650, 390, and 140 W/cm^2^ (PNP = 4, 3, and 2 MPa), respectively. (*n* ≥ 4, mean ± S.D.). The arrowed bracket identifies this transformation point for an acoustic intensity of 140 W/cm^2^.

The maximum PFC nanodroplet concentration appropriate for a given HIFU intensity was selected based on the results of evaluating the vaporization field (microbubble cloud) shape and location across a broad range of concentrations (10^5^ to 10^8^ ND per milliliter) treated by continuous wave HIFU for 20 s at 2, 3, and 4 MPa (intensities of 140, 390, and 650 W/cm^2^). Figure 3 shows the progressive shape and location changes observed with increasing PSNE concentrations: cigar surrounding the central acoustic focal point, tadpole with prefocal migration, and eventually flattened microbubble clouds along the frontal surface. These changes result from vaporization concentrating in prefocal areas and are associated with a risk of ablation occurring away from the intended target site. Although flattening of the microbubble clouds along the prefocal phantom surface was an artifact of the layered gel model, their appearance was nevertheless a clear indication that control over the placement of the vaporization field had been lost. The transformations in microbubble cloud shape (distortion) occurred on a continuum, as reflected by the logarithmic relationship between the lateral axis lengths and PSNE concentration for a given acoustic intensity (*R*^2^ ≥ 0.94, *p* < 0.001, linear regression on log scale) (Figure 4A). This cigar to tadpole transformation was reflected by a statistically significant (*p* ≤ 0.01) change in the area-to-perimeter ratio (Figure 4B) with increasing PSNE concentration. The point of initial distortion corresponded with a statistically significant (*p* ≤ 0.03) center shift (Figure 4C). For the range of PSNE concentrations and acoustic intensities tested, distortion and center shift increased with increasing intensity for a given concentration and also occurred at lower PSNE concentrations in response to higher acoustic intensities (Figure 4B,C).

Tadpole transformations began at PSNE concentrations of 5 × 10^5^, 2.5 × 10^6^, and 1 × 10^7^ ND per milliliter (0.005%, 0.025%, 0.1% *v*/*v*) using intensities of 650, 390, and 140 W/cm^2^, respectively. These concentration-intensity combinations were chosen for further study, since they were the maximum points at which predominately cigar-shaped ablation lesions could still be found after 20 s of insonation. Of note, these intensities and PSNE concentration ranges are similar to those reported previously [20].

### Vaporization field volume as a function of the HIFU treatment cycle

**Figure 5:**
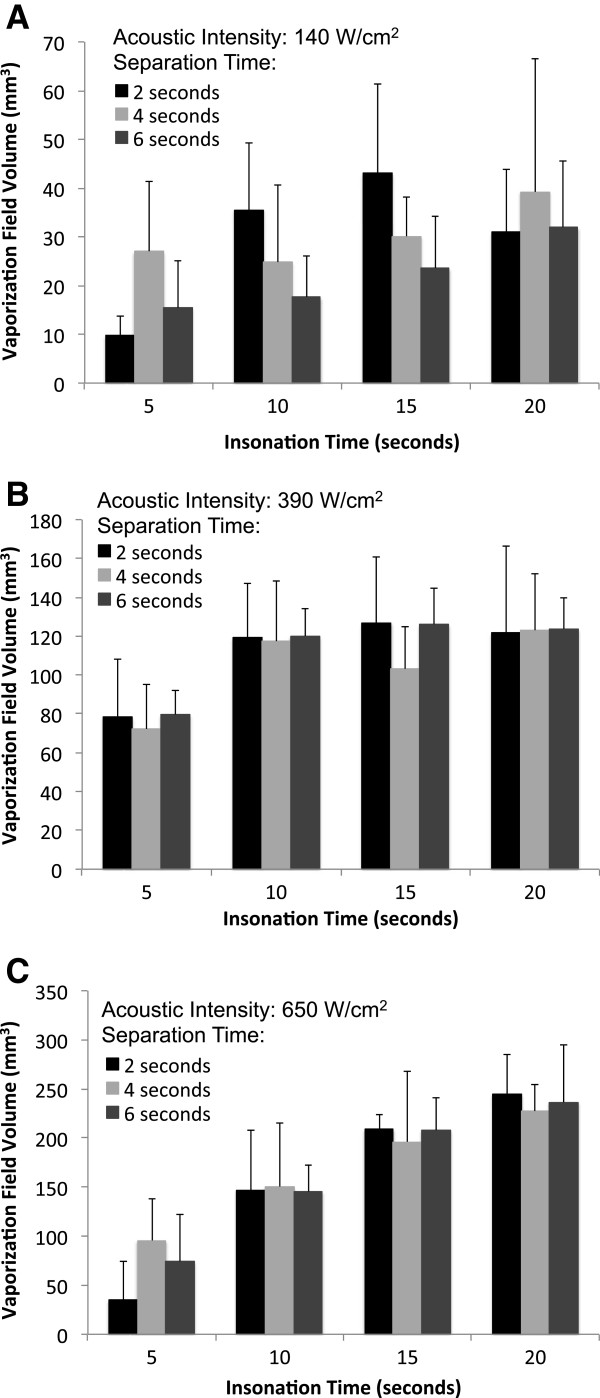
**Relationships between the volume of the vaporization field, separation time, and insonation time.** At intensities of **(A)** 140, **(B)** 390, and **(C)** 650 W/cm^2^, no statistically significant differences were observed between the volumes of the vaporization fields at separation times of 2, 4, and 6 s for any given insonation time (*n* ≥ 4, mean ± S.D.).

The treatment cycle of continuous wave HIFU refers to the insonation (‘on’ or ablation) time and the ‘off’ or cool-down time that separates the insonation times. For a given intensity, there was no statistically significant difference in the volumes of the vaporization fields (microbubble clouds) when insonation was separated by ‘off’ times lasting 2, 4, or 6 s. This consistency was present across the entire range of insonation times (Figure 5).

**Figure 6:**
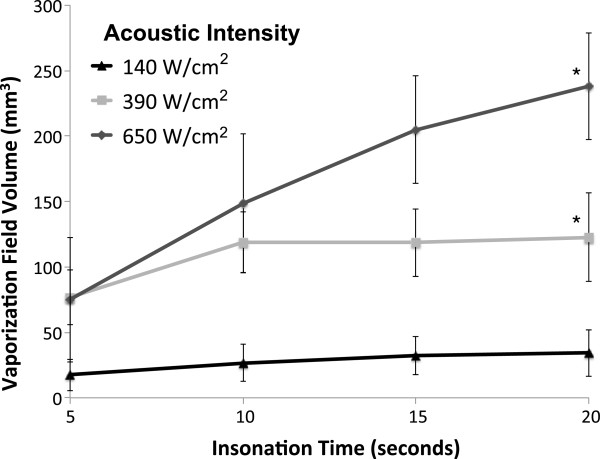
**Volume of the vaporization field (microbubble cloud) as a function of the duration of insonation.***n* ≥12, mean ± S.D., *p* < 0.02 for values along each intensity curve (ANOVA), Single asterisk (*) indicates significance (*p* < 0.008) between the volumes of the vaporization fields at insonation times of 5 and 20 s for the given acoustic intensity (Bonferroni correction to Student's *t* test).

Given the fact that varying the separation times did not affect the vaporization volume, all measurements collected for a given insonation time were averaged for further analysis. There was a statistically significant dose-responsive relationship (ANOVA, *p* < 0.02) between the duration of HIFU insonation and the volumes of the resulting vaporization fields (Figure 6). The volumes of the vaporization field ranged from approximately 75 to 240 mm^3^ with the application of HIFU at 650 W/cm^2^ from 5 to 20 s, respectively, in phantoms containing 5 × 10^5^ ND per milliliter. These fields were much smaller when 140 W/cm^2^ was applied to phantoms containing 1 × 10^7^ ND per milliliter, ranging from approximately 15 mm^3^ after 5 s of insonation to 25 mm^3^ after 20 s. Additionally, at this lowest acoustic intensity, the vaporization response was inconsistent, demonstrating that this is the minimum intensity required to achieve vaporization using this PSNE.

Bubble fields were not seen by conventional acoustic imaging in the agent-free phantoms when HIFU was applied at intensities less than 650 W/cm^2^. At 650 W/cm^2^ for 20 s, small bubble fields averaging <4 mm^3^ are formed. These areas were most likely boiling clouds generated from dissolved gases in the phantom gel.

### Ablation lesion size as a function of the HIFU treatment cycle

**Figure 7:**
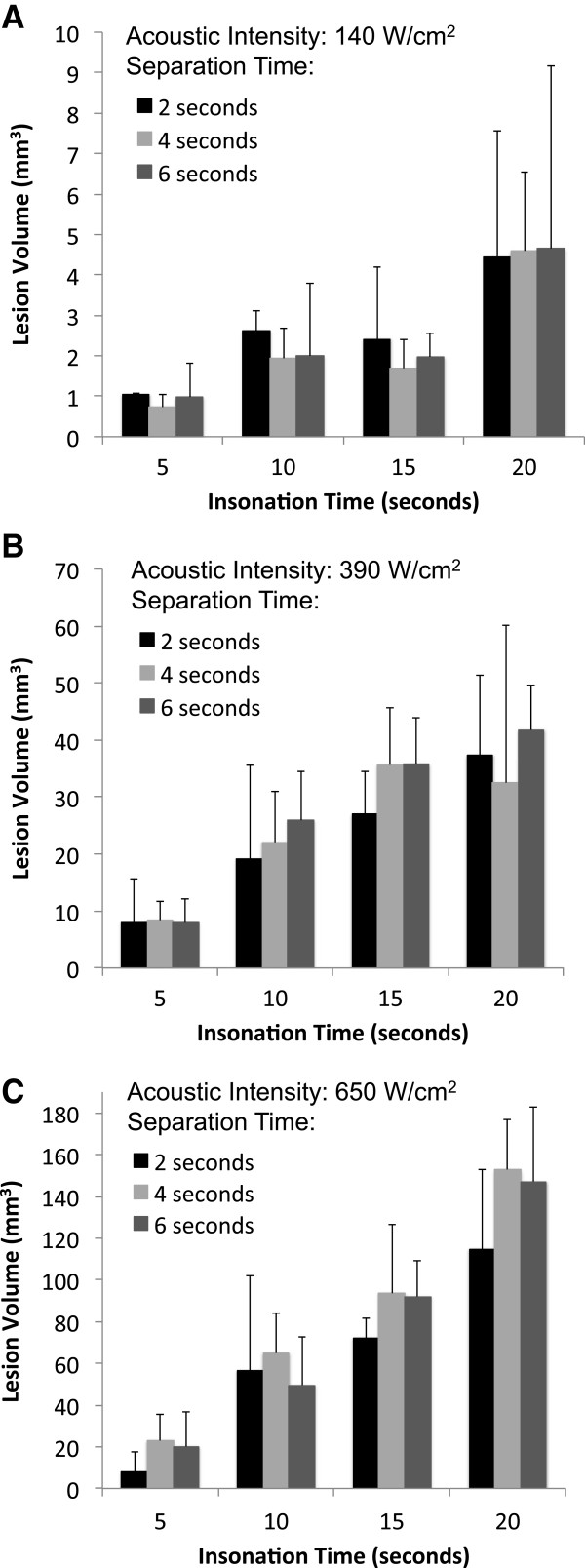
**Ablation lesion size as a function of the separation time of the HIFU treatment cycle.** At intensities of **(A)** 140, **(B)** 390, and **(C)** 650 W/cm^2^, no statistically significant differences were observed between the volumes of the ablation lesions at separation times of 2, 4, and 6 s for any given insonation time (*n* ≥ 4, mean ± S.D.).

**Figure 8:**
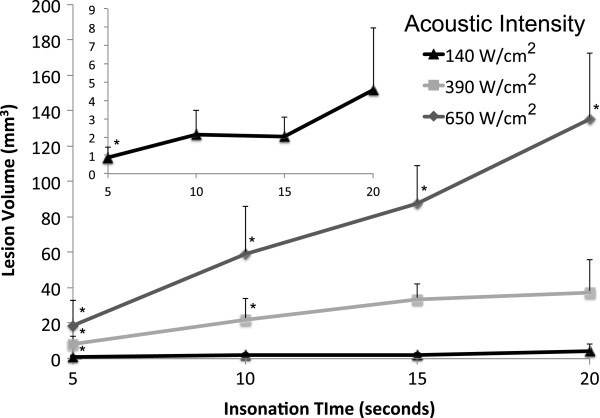
**Ablation lesion size as a function of the duration of insonation (‘on’ or ablation time).** The inset shows the lowest intensity data in detail. *n* ≥ 12, mean ± S.D., *p* < 0.001 for values along each intensity curve (ANOVA), single asterisk (*) indicates significance (*p* < 0.008) from all other insonation times for the given acoustic intensity (Bonferroni correction to Student's *t* test).

Varying the ‘off’ or cool-down time that separated ablative insonation by 2, 4, and 6 s did not affect the volume of the ablation lesion for a given intensity and duration of insonation (Figure 7). However, a statistically significant dose responsive relationship (ANOVA, *p* < 0.001) was observed between the insonation time and the volume of the resulting ablation lesion (Figure 8). Averaging all measurements collected at a given insonation time demonstrated this relationship to be present even at the lowest acoustic intensity of 140 W/cm^2^ (PNP = 2 MPa), although the resulting ablation lesions were small and appeared inconsistently. On average, these ablation lesions measured just 4 mm^3^ after 20 s of HIFU, despite the fact that the phantoms contained the highest PFC concentration (1 × 10^7^ ND per milliliter). This finding again demonstrated that 140 W/cm^2^ should be considered the minimum required intensity to achieve controllable ablation with this PSNE. When higher intensities were applied, the ablation lesions were significantly larger, even though the PFC concentrations were lower. Phantoms containing 2.5 × 10^6^ ND per milliliter received HIFU at an intensity of 390 W/cm^2^ (PNP = 3 MPa). The resulting ablation lesions measured 8 mm^3^ after 5 s and 37 mm^3^ after 20 s. Applying an intensity of 650 W/cm^2^ (PNP = 4 MPa) to phantoms containing 5 × 10^5^ nanodroplets per milliliter resulted in ablation lesions ranging from 19 to 135 mm^3^ with insonation times from 5 to 20 s.

Minimal ablation occurred in phantoms containing no PFC agent. The average ablation lesion in response to the maximum energy delivered during these experiments [20 s of continuous wave HIFU at 650 W/cm^2^ (PNP = 4 MPa)] measured 3 mm^3^, and the appearance of these lesions was inconsistent.

### Optimizing acoustic parameters for a given PFC concentration

**Figure 9:**
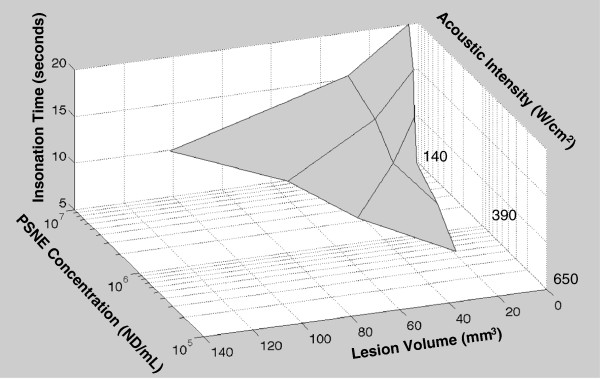
**Relationships between acoustic intensity, insonation time, PFC nanodroplet concentration, and ablation lesion size.** This three-dimensional grid demonstrates the best acoustic parameters (intensity and insonation time) to produce an ablation lesion of desired size in a region containing a known agent concentration for this specific PSNE.

The results of this study demonstrate that the size of an ablation lesion produced during PFC-enhanced HIFU can be predicted *in vitro* from the acoustic intensity, the duration of insonation, and the concentration of the PFC agent. This level of control would theoretically allow selection of the best acoustic parameters (intensity and insonation time) to produce an ablation lesion of desired size in a region of known PFC concentration. These relationships can be visualized by a three-dimensional plot relating acoustic intensity, insonation time, PFC nanodroplet concentration, and ablation lesion size (Figure 9). It should be noted that these quantified relationships apply only to this specific PSNE, although it is reasonable to assume that similar relationships could be defined for other PSNEs.

### Comparing vaporization field volumes and the sizes of the ablation lesions within them

Both the volume of the vaporization field (microbubble cloud) and the volume of the ablation lesion within the microbubble cloud increased with increasing insonation times (Figures 6 and 8). The vaporization clouds were larger than the ablation lesions contained within them. After 5 s at 650 W/cm^2^, the vaporization field was four times larger than its ablation lesion. However, with increasing insonation time, ablation occurred in a larger portion of the vaporization field. For example, after 20 s at 650 W/cm^2^, this ratio was down to 1.75. Similarly, ablation filled more of the microbubble cloud when higher intensities were used. After 20 s of HIFU at 140 W/cm^2^, the vaporization field was about 7.5-fold larger than the ablation lesion within it, compared to 1.75-fold at 650 W/cm^2^. These trends probably, at least in part, reflected the containment of the vaporization field within the gel boundaries that are present in this experimental setup. However, the fact that ablation fills more of the microbubble cloud with higher intensities and insonation times also suggested that vaporization and ablation grew outward from the central focal zone throughout the continuous wave insonation.

## Discussion

PFC microbubbles act as cavitation nuclei and lower the acoustic intensity required to achieve ablation by HIFU. Lowered energy requirements should improve HIFU safety and make it a viable option to treat a broader range of disease sites. Vaporizing liquid PFC droplets in the target area provides an attractive option for introducing these microbubbles, given the stability of PFC droplets in circulation and the fact that they can be manufactured small enough (<300 nm) to extravasate through the leaky tumor vasculature. Previous testing has confirmed that phase-shift agents using several different PFC formulations produce larger ablation lesions with lower HIFU energies [9,17,20]. However, clinical utility requires control over the size, shape, and location of the ablation lesion, and this control can be problematic when PFC agents are present in the acoustic field [9,11,20,21], particularly if the PSNE includes a volatile PFC component.

This study explored the use of a PSNE containing highly volatile DFB (1:1 mix of DFB and DDFP) in a gel model of HIFU enhancement. Lowering the intensity needed to achieve vaporization by the addition of a volatile PFC component can minimize HIFU intensity requirements. However, since the acoustic pressure that triggers vaporization is similar to the pressure that drives cavitation, establishing a vaporization field that does not grow during lower amplitude continuous wave insonation is not possible. Therefore, these experiments focused on optimizing acoustic parameters (intensity and insonation time) to achieve controllable HIFU enhancement for a given concentration of this novel PSNE. Previous testing has defined the limits of effective acoustic intensity for this PSNE [9]. Even when PSNE was present, no ablation was seen at intensities below 140 W/cm^2^, and ablation in agent-free gels started to occur above 650 W/cm^2^. Therefore, the current study explored the effects of varying insonation (‘on’) and separation (‘off’) times of the HIFU treatment cycle within the intensity range from 140 to 650 W/cm^2^. It should be noted that this intensity range corresponds well with the range (157 to 550 W/cm^2^) identified by Zhang P. et al. as most appropriate for PFC-enhanced HIFU [20]. In contrast, current HIFU systems in use clinically must operate at intensities at or above 2,000 W/cm^2^, risking injury to tissue outside of the target site. This study demonstrated that appropriate acoustic intensities and insonation times can be selected to maintain control over ablation lesion size, shape, and location even in the presence of a PSNE containing a volatile PFC component. Additionally, since the vaporization field was not fixed, relatively large ablation lesions that propagated outward from the acoustic focus could be produced. The ablation lesions measured up to 135 mm^3^ in gels with PSNE, compared to lesions measuring <4 mm^3^ in agent-free gels, when HIFU was applied at 650 W/cm^2^ for 20 s.

There are interesting similarities and differences between these findings and the results of other phase-shift studies looking at HIFU enhancement by PFC nanodroplets and microdroplets in acrylamide gels. Using a DDFP-based PSNE without a more volatile component, Zhang P. et al. were also able to produce ablation lesions larger than 100 mm^3^. However, the required intensities (around 2,000 W/cm^2^) were higher [20]. It is suspected that the differences in ablation response between these two studies resulted primarily from the presence of volatile DFB in our PSNE. Testing in solution has demonstrated that incorporating a lower boiling point PFC during the PSNE preparation decreases the vaporization threshold of the PSNE [16]. As a result of this lower threshold, the vaporization field increased in volume throughout the continuous wave HIFU treatment cycle in our study (Figure 6). In contrast, in the presence of DDFP alone, Zhang P. et al. established a stable vaporization field using a 30-cycle high-intensity (4,586 W/cm^2^) pulse and followed this with 15 s of continuous wave HIFU [20]. Additionally, it should be noted that the frequency of applied HIFU was also different in these two studies (1 vs. 3.2 MHz). PFC droplet vaporization is a frequency-dependent phenomenon, and frequency differences alter ultrasound wave attenuation, energy deposition, and microbubble cavitation [29].

Zhang M. et al. studied DDFP microdroplet enhanced HIFU delivered at a frequency (1.44 MHz) similar to ours [21]. They too noted increasing ablation lesion volumes with longer insonation times (insonation duration 2 to 5 s). However, at comparable PFC agent concentrations (3 vs. 5 × 10^5^ droplets per milliliter) and insonation duration (5 s), the ablation responses were quite different. Probably reflecting the use of larger PFC droplets (2 to 8 microns) and a higher intensity (4,000 W/cm^2^), Zhang M. et al. produced ablation lesions measuring 600 mm^3^, compared to 20 mm^3^ in our study [21].

Finally, again testing a DDFP PSNE in acrylamide gels, Kawabata et al. applied HIFU using acoustic parameters (frequency, 1.1 MHz; intensity, 600 W/cm^2^; insonation duration, 5 to 20 s) similar to our study and found increasing ablation lesion size with longer insonation [29]. However, lesion size did not increase when HIFU was applied at 2.2 MHz. The similarities and differences between these studies emphasize the fact that the ablation response during PSNE enhanced HIFU results from a complex interplay of the applied acoustic parameters as well as the type and size of the PFC agent. As a result, it is suspected that changing the ratio of DFB and DDFP in our PSNE would alter both the vaporization and ablation responses and is a potential area of future study.

The ablation lesions formed in this study were located in larger vaporization fields (microbubble clouds). The fact that the vaporization field grows throughout the entire HIFU treatment cycle when a volatile PSNE is present distinguishes this study from previous phase-shift enhancement studies. Continuous vaporization provides an opportunity to generate larger ablation lesions but also introduces unique challenges with regard to controlling the ablation response. Anticipating the extent of vaporization surrounding an ablation site is of practical importance when positioning adjacent lesions. Separating insonation by 2, 4, or 6 s did not affect the size of the vaporization field or the ablation lesion within this microbubble cloud when adjacent HIFU sites were 8 mm apart. Decreasing this separation distance could result in overlapping vaporization fields. Future study will be needed to assess the impact of overlapping vaporization fields on tissue heating and the ablation response.

In this gel model, controlled ablation could be achieved using acoustic parameters that did not result in lesion formation in agent-free regions (frequency = 1 MHz, intensity ≤ 650 W/cm^2^, insonation time ≤ 20 s). However, the limitations of this model should be noted. The phantoms and surrounding gels were acellular and avascular. Therefore, tissue injury that would result from mechanical cavitation effects was not captured, and heat sink effects of blood flow were not considered. Also, changes in acoustic impedance due to trapped degassed water between the acrylamide gel cap, the PFC-containing phantom, and the gellan gum mold resulted in echogenic boundaries, which may have slightly attenuated transmitted energies. Such distinct separations between agent-free and PFC-containing tissue regions would not be seen *in vivo* nor would the uniform distribution of PFC agent achievable *in vitro*.

A PSNE concentration in the range of 10^5^ to 10^7^ ND per milliliter was required for enhanced ablation in this gel model. Further experimentation will be needed to determine how these concentrations can be achieved *in vivo*. Recent testing has shown that the intravenous administration of a DDFP-based PSNE (0.5 mL/kg) resulted in the preferential deposition of nanodroplets in the vascular periphery of a rabbit tumor [17]. These agents remained stable for hours and accumulated to a concentration sufficient to lower the energy required to reach an ablative temperature by HIFU. The DDFP PSNE used in this *in vivo* study was also tested *in vitro* by Zhang P. et al. in concentrations similar to those used in our current study [20]. It is therefore reasonable to assume that effective tissue concentrations of our DFB-DDFP PSNE can also be achieved. However, to date, tissue concentrations of PFC nanodroplets following the administration of a PSNE have not been quantified *in vivo*. Magnetic resonance of fluorine or gadolinium-tagged agents may provide a tool to measure PFC tissue concentrations [20,30]. The development of such a tool may be an important step on the path toward using PSNEs clinically for several reasons. First, there is a heterogeneous deposition of PFC in tumor tissue following the intravenous administration of a PSNE [17], quite different from the uniform distribution used for *in vitro* studies. Second, as this and other studies have now demonstrated, controlled ablation requires selecting appropriate acoustic parameters for a given PSNE concentration. Therefore, the safe application of HIFU in a field containing an unequal distribution of PFC agent may require estimating regional PFC concentrations and adjusting the acoustic parameters accordingly. We envision a magnetic resonance-guided focused ultrasound surgery system that maps the distribution of PFC tissue levels, selects appropriate acoustic parameters for the target region and desired lesion size, and monitors ablation by thermometry.

## Conclusions

Research in this and other laboratories has now validated the use of phase-shift PFC nanoemulsions to enhance HIFU by seeding the acoustic target area with potential cavitation sites. Several methods are available to synthesize nanodroplets that are stable to circulate at body temperature and small enough to extravasate into interstitial tissue spaces. Additionally, appropriate nanodroplet concentrations and acoustic parameters are being defined to achieve enhancement (larger ablation lesions using shorter treatment cycles and lower acoustic power) while maintaining control over lesion shape and location. This *in vitro* study used a unique PSNE containing a volatile PFC component and found that enhanced ablation could be achieved with acoustic intensities low enough to avoid ablation in agent-free regions. However, control over the ablation response was problematic, unless specific acoustic parameters were selected for the available PSNE concentration. These findings support continued experimentation using PSNEs to enhance HIFU but also emphasize the importance of exploring tools to quantify PFC tissue concentrations *in vivo* to guide acoustic parameter selection.

## Abbreviations

DDFP: Dodecafluoropentane; DFB: Decafluorobutane; HIFU: High-intensity focused ultrasound; ISP: Spatial peak intensity; ND: Nanodroplets; PFC: Perfluorocarbon; PNP: Peak negative pressure; PSNE: Phase-shift nanoemulsion.

## Competing interests

The authors declare that they have no competing interests.

## Authors' contributions

CP carried out the experiments, analyzed the data, and drafted the manuscript. LCP participated in the study design and helped to revise the manuscript. PSS prepared the perfluorocarbon nanoemulsions and helped revise the manuscript. PAD participated in the study design and coordination and edited the manuscript. All authors read and approved the final manuscript.
